# Evidence of a Critical Role for Cellodextrin Transporte 2 (CDT-2) in Both Cellulose and Hemicellulose Degradation and Utilization in *Neurospora crassa*

**DOI:** 10.1371/journal.pone.0089330

**Published:** 2014-02-20

**Authors:** Pengli Cai, Ruimeng Gu, Bang Wang, Jingen Li, Li Wan, Chaoguang Tian, Yanhe Ma

**Affiliations:** 1 Key Laboratory of Systems Microbial Biotechnology, Tianjin Institute of Industrial Biotechnology, Chinese Academy of Sciences, Tianjin, China; 2 Institute of Microbiology, Chinese Academy of Sciences, Beijing, China; Oregon State University, United States of America

## Abstract

CDT-1 and CDT-2 are two cellodextrin transporters discovered in the filamentous fungus *Neurospora crassa*. Previous studies focused on characterizing the role of these transporters in only a few conditions, including cellulose degradation, and the function of these two transporters is not yet completely understood. In this study, we show that deletion of *cdt-2*, but not *cdt-1*, results in growth defects not only on Avicel but also on xylan. *cdt-2* can be highly induced by xylan, and this mutant has a xylodextrin consumption defect. Transcriptomic analysis of the *cdt-2* deletion strain on Avicel and xylan showed that major cellulase and hemicellulase genes were significantly down-regulated in the *cdt-2* deletion strain and artificial over expression of *cdt-2* in *N. crassa* increased cellulase and hemicellulase production. Together, these data clearly show that CDT-2 plays a critical role in hemicellulose sensing and utilization. This is the first time a sugar transporter has been assigned a function in the hemicellulose degradation pathway. Furthermore, we found that the transcription factor XLR-1 is the major regulator of *cdt-2*, while *cdt-1* is primarily regulated by CLR-1. These results deepen our understanding of the functions of both cellodextrin transporters, particularly for CDT-2. Our study also provides novel insight into the mechanisms for hemicellulose sensing and utilization in *N. crassa*, and may be applicable to other cellulolytic filamentous fungi.

## Introduction

Lignocellulose degradation is a critical step for biofuels and bio-based chemical production in biorefineries [1], [2]. The mechanism by which cellulolytic organisms, such as fungi, sense and metabolize solid cellulose and hemicellulose is still far from understood. Typically, lignocellulases are expressed at a basal level on the periphery of conidia, and these enzymes degrade biomass into various carbohydrates [3], [4]. These carbohydrates include: cellodextrins (glucose polymers, such as cellobiose, cellotriose and cellotetraose) derived from cellulose, and xylodextrins (xylose polymers, such as xylobiose, xylotriose and xylotetraose) derived from hemicellulose [5]–[7]. Oligosaccharides such as cellodextrins and xylodextrins are thought to function as inducer molecules. Cellodextrin and its modifiers have been implicated as cellulase inducers in *Trichoderma reesei* [3], [8] and *Neurospora crassa* [9]. Xylobiose was suggested as a xylanase inducer in the thermophilic fungus *Thermoascus aurantiacus* [10]. It is possible that organisms sense cellulose or hemicellulose through recognition of these oligosaccharides by a transporter in the membrane, and in fact the genome of *N. crassa* encodes for two cellodextrin transporters, *cdt-1* and *cdt-2* [11]. In addition, two major facilitator superfamily (MFS) sugar transporters, Stp1 and Crt1, were implicated in cellulose sensing and cellulase induction in *T. reesei* [12]. Stp1 repressed the induction of cellulases and hemicellulases on Avicel, while the Crt1 is required for cellulase induction by cellulose, lactose, and possibly sorphorose. However, Crt1 was not required for hemicellulase expression on xylan [12], [13]. Similarly, two cellodextrin transporters, CdtC and CdtD, were identified in *Penicillium oxalicum*, and these transporters affect cellulase induction and cellulose utilization [14]. Sugar transporters involved in sensing hemicellulose have not been previously reported.

CDT-1 and CDT-2 were the first cellodextrin transporters identified in filamentous fungi, and it was previously shown that deletion of *cdt-2* caused significant growth defects on cellulose, while the *cdt-1* deletion strain showed similar growth to that of wild type [11]. An engineered *Saccharomyces cerevisiae* strain expressing *cdt-2* exhibited lower rates of cellobiose fermentation compared with engineered strains carrying *cdt-1* [15]. These results suggest that although they have redundant roles in cellobiose transport, there are differences between the functions of the *cdt-1* and *cdt-2* [11]. The function of *cdt-1* and *cdt-2* has only been evaluated with respect to cellulose degradation (Avicel, cellobiose) [11]. Additional conditions, such as growth on hemicellulose (the second major part of the plant biomass), need to be investigated to fully characterize the role of these two cellodextrin transporters.

Very little is known about the regulation of *cdt-1* and *cdt-2*, although cellulose and hemicellulose degradation regulators can affect their expression. In previous research, *cdt-1* and *cdt-2* expressions were modulated in mutants of *clr-1* or *clr-2*, two major cellulose degradation regulators in *N. crassa* [16]. This data suggested that both cellodextrin transporters could be regulated by CLR-1 and CLR-2, either direct or indirectly. XlnR and its orthologs are conserved lignocellulase regulators, particularly for hemicellulase expression in filamentous fungi, including XlnR in *Aspergillus niger* [17], XYR-1 in *T reesei* [18] and XLR-1 in *N. crassa* [19]. Transcriptional profiling of the *xlr-1* mutant in *N. crassa* identified *cdt-2* as one of 245 genes in the putative XLR-1 regulon [19].

In the present study, we comprehensively investigated the function of *cdt-1* and *cdt-2* by assessing the phenotypes of single and double knock-outs of these genes under a variety of carbon conditions, including growth on hemicellulose. We also evaluated the expression and localization patterns of these transporters under cellulose and hemicellulose conditions, and conducted a transcriptomic analysis of the *cdt-2* deletion mutant in both cellulose and hemicellulose conditions. Higher cellulase and hemicellulase production were observed in strains artificially over-expression *cdt-2*. Finally, we performed a comparative analysis of the transcriptional regulation of *cdt-1* and *cdt-2*. This is the first analysis of cellodextrin transporter function with regards to hemicellulose degradation and utilization. Our results indicated that CDT-2 plays critical roles in both sensing and utilization of cellulose and hemicellulose, and provide novel insights that can be applied to cellulase and hemicellulase production for industrial fungi.

## Materials and Methods

### Strains and Culture Conditions

All strains used in this study are listed in Table 1. *N. crassa* strains were obtained from the Fungal Genetics Stock Center (FGSC) [20], including wild type (WT, FGSC 2489), two cellodextrin transporter deletion strains (FGSC 16575, Δ*cdt-1*; FGSC 17868, Δ*cdt-2*) and a *his-3* mutant strain (FGSC 6103, *his-*3). The double deletion strain Δ*cdt-1*Δ*cdt-2* resulted from a cross between Δ*cdt-1* and Δ*cdt-2* using previously described methods [21]. Strains Pc-*cdt-2* and Pn-*cdt-2* were the Δ*cdt-2* complemented strains carrying either the *ccg-1* promoter (Pc) or the native promoter (Pn) of *cdt-2*. The complemented strains were constructed by transforming the plasmid pMF272-*Pccg-1*-*cdt2*-GFP and pMF272-native-*cdt2*-GFP into Δ*cdt-2 his-3*, which was obtained from a cross between Δ*cdt-2* and a *his-3* mutant strain. All of the constructed *N. crassa* strains were verified by PCR (Figure S1 in File S1).

**Table 1:** Strains and plasmids used in this study.

Strain	Genotype/comment	source
*N. crassa*		
wild type	FGSC2489	FGSC
Δ*cdt-1*	FGSC 16575, *cdt-1* deletion strain	FGSC
Δ*cdt-2*	FGSC 17868, *cdt-2* deletion strain	FGSC
Δ*cdt-1*Δ*cdt-2*	Double deletion strain of *cdt-1* and *cdt-2*	This study
*his-*3	FGSC 6103, *his-3* mutant strain	FGSC
Δ*cdt-2 his-3*	Double deletion strain of *his-3* and *cdt-2*	This study
Pc-*cdt-2*	*his-3*::pMF272-*Pccg-1*-*cdt2*-GFP; Δ*cdt-2 his-3*	This study
Pn-*cdt-2*	*his-3*::pMF272-native-*cdt2*-GFP; Δ*cdt-2 his-3*	This study
CPL-1	*his-3*::pMF272-*Pccg-1*-*cdt2-*GFP; *his-3*	This study
plasmids		
pMF272-*Pccg-1*-GFP	*N. crassa* GFP tagging vector with *ccg-1* promoter	[43]
pMF272-*Pccg-1*-*cdt2*-GFP	pMF272-*Pccg-1*-GFP with cDNA of *cdt-2*	[44]
pMF272-native-*cdt2*-GFP	pMF272-GFP with cDNA of *cdt-2* and *cdt-2* native promoter	This study

To obtain conidia, *N. crassa* was grown on slant tubes containing Vogel’s minimal media with 2% (w/v) sucrose (MM) for 10 days at 28°C. The 50×Vogel’s salts was prepared as previously described [22]. For liquid cultures, *N. crassa* was cultivated in 100 ml Vogel’s salts with different carbon sources at 2% (w/v). The carbon sources included glucose, xylose, cellobiose, xylodextrin (cat. no. 245-00751), Avicel PH-101, and xylan (Birchwood, cat. no. X0502). All carbon sources were purchased from Sigma-Aldrich, except xylodextrin, which was obtained from Wako. 100 ml cultures were inoculated with 10^6^ conidia per ml. For biomass measurement, all cultures were grown at 25°C for 1–3 days at 200 rpm. For gene expression level measurement, the *N. crassa* wild type strains were cultured in 2% (w/v) glucose, cellobiose, xylodextrin and xylan for 16 h, xylose for 22 h and Avicel for 30 h respectively in order to obtain cultures with similar hyphal development.

### Complementation of Δcdt-2 and Subcellular Localization of CDT-2-GFP in *N. Crassa*

All plasmids and primers used in this study are listed in Table 1 and Table S1, respectively. *cdt-2*, with a 1000-bp upstream region, was PCR amplified from wild-type *N. crassa* genomic DNA using the primers ΔCDT2-F-N and ΔCDT2-R-N. After digestion with *Not*I and *Pac*I, the fragment was inserted into plasmid pMF272, which carries GFP next to the multiple cloning sites. The resulting plasmid was designated as pMF272-native-*cdt2*-GFP. The plasmid pMF272-*Pccg-1*-*cdt2*-GFP was a kind gift from the laboratory of Prof. Louise Glass from U. C. Berkeley [23]. The resulting plasmids were transformed into strain Δ*cdt-2 his-3*, which was obtained from a cross between Δ*cdt-2* and *his-3* strains. The transformation was conducted according to Vann [24]. The resulting complemented strains were named Pc-*cdt-2* and Pn-*cdt-2*. For biomass measurement, the strains were cultured in 100 ml Vogel’s salts containing 2% (w/v) xylan for 3 days. For gene expression analysis, the strains were cultured in 100 ml liquid MM media for 16 h at 25°C. Mycelia were collected and washed with Vogel’s salts. Subsequently, the mycelia were transferred into 100 ml Vogel’s salts containing 0.5% (w/v) xylan and incubated for an additional 4 h. The mycelia were harvested, and the RNA extraction was performed as previously described [25].

To localize GFP fusion proteins using microscopy, all strains were inoculated in liquid MM medium and grown for 16 h. The hyphae were harvested, washed with Vogel’s salts and transferred into inducing media containing 0.5% (w/v) Avicel or xylan. The cultures were incubated for an additional 4 h at 25°C. Before imaging, the hyphae were incubated with 1 µg/ml DAPI for 15 min. The microscopic observation was performed on a Laser Scanning Confocal Microscope Leica TCS SP5 II (Leica), and Leica Microsystems LAS AF-TCS MP Version: 2.4.1 build 6384 and ImageJ software were used for image processing.

### Xylodextrin Consumption Assays in *N. Crassa*

Wild type, Δ*cdt-2* and Pc-*cdt-2* strains were grown for 16 h in 100 ml liquid MM at 25°C. The mycelia were harvested through centrifugation at 4°C and 3500×g, washed three times with Vogel’s salts, and subsequently transferred to Vogel’s salts containing 0.5% (w/v) xylan for an additional 4 h of inducing cultivation. A total of 10 ml of the culture was harvested through centrifugation at 4°C and 3500×g, washed three times with Vogel’s salts, and resuspended in 1 ml of double distilled water containing cycloheximide (100 µg/ml) and either 100 µl xylobiose or xylotriose (10 mM). The mycelia were removed through centrifugation after 15 min to measure xylobiose or xylotriose consumption. The amount of sugar remaining in the supernatant was determined by HPLC (Waters e2695 separations Module) with Aminex HPX-87H and Aminex HPX-87P columns (Bio-Rad). The peaks were detected using a Waters 2414 refractive index detector, and 1,4-β-D-xylobiose and 1,4-β-D-xylotriose were purchased from Megazyme (Bray, Ireland).

### Biochemical Methods

The total extracellular protein content was determined using the Bradford method, with BSA as the standard [26]. The mycelia of 100 ml liquid cultures were collected through vacuum filtration after 3 days of cultivation, enclosed with tin foil, and dried for 17 h at 110°C in a drying oven. The dry weight of the mycelia was measured to determine the biomass. The endoglucanase activity was measured using an azo-CMC kit (S-ACMCL). The xylanase and β-xylosidase activities were measured using a previously published method [27].

### RNA-seq and Data Analysis

The fungus was inoculated into 100 ml of liquid MM media to a spore concentration of 10^6^ conidia per ml, and the cultures were grown for 16 h at 25°C. The mycelia were collected, washed with Vogel’s salts, and subsequently transferred into 100 ml Vogel’s salts with 0.5% (w/v) carbon source (xylan or Avicel) for an additional 4 h of cultivation. The mycelia were collected through filtration and immediately frozen in liquid nitrogen. The RNA was extracted as previously described [25]. Total RNA was treated with DNase I (Turbo DNA-free kit; Ambion), and the RNA was subsequently used for either RNA-seq or qRT-PCR experiments.

mRNA sequencing was performed at BGI (Shenzhen, China). The sequenced libraries were mapped against the *N. crassa* OR74A genome (version 12) with less than two-base mismatching, using Tophat (version 2.0.8b) [28]. The alignment results were stored in SAM format files for subsequent analysis. Read counts uniquely mapped to only one gene in predicted transcripts (version 7) were calculated for every individual gene using HTseq-count (http://www-huber.embl.de/users/anders/HTSeq) using SAM files and genome annotation as input. The normalized expression values for each gene were calculated using the number of uniquely mapped reads per kilobase of exon region per million mapped reads (RPKM). DEGseq software was used to identify the differentially expressed genes for RNA-seq data from different samples, the P-value was determined by Fisher’s exact test and Likelihood ratio test [29]. The three raw RNA-seq data sets generated in this study (WT exposure to xylan for 4 h, *cdt-2* mutant exposure to Avicel for 4 h, and *cdt-2* mutant exposure to xylan for 4 h) are available in the GEO database (GSE44673; http://www.ncbi.nlm.nih.gov/geo/). The RNA-seq data of WT exposed to Avicel for 4 h was downloaded from NCBI (GSE36719) [9], and the RNA-seq data of WT exposure to no carbon for 4 h was downloaded from NCBI (GSE35227) [16].

### Overexpression of cdt-2 in *N. crassa*

For *cdt-2* overexpression in *N. crassa*, the plasmid pMF272-*Pccg-1*-*cdt2*-GFP was transformed into a wild type *his-3* strain (FGSC 6103). This *cdt-2* overexpression strain was designated as CPL-1. For the gene expression analysis, the wild type strain and CPL-1 were pre-cultured in MM for 16 h and transferred to inducing conditions (2% Avicel or 2% xylan) for an additional 24 h of cultivation. For enzyme activity measurements (endoglucanase activity, xylanase activity and β-xylosidase activity), the wild type strain and CPL-1 were cultured for 5 days in 2% (w/v) Avicel or 2% (w/v) xylan medium.

### Real-time Quantitative Reverse Transcription PCR

Real-Time quantitative Reverse Transcription PCR (qRT-PCR) was performed through two-step RT-PCR using the iScript cDNA Synthesis Kit and IQ SYBR Green Supermix according to the manufacturer’s instructions (Bio-Rad). The primers used are listed in Table S1. Each reaction was done in triplicate. Actin expression (NCU04173) was used as an endogenous control for normalization as previously described [30].

## Results

### Δcdt-2, but not Δcdt-1, Shows a Severe Growth Defect on Hemicellulose

**Figure 1:**
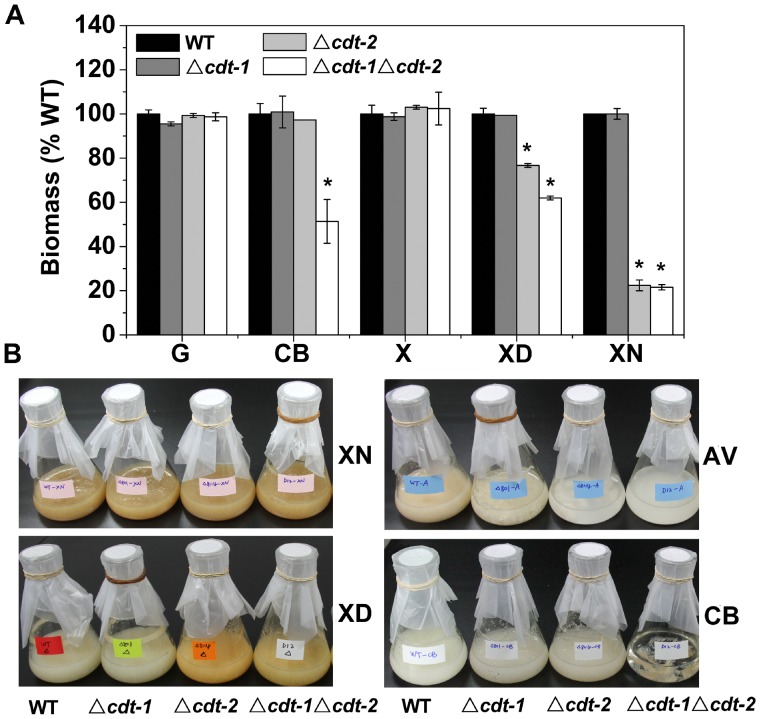
Growth phenotypes of *cdt* mutants on different carbon sources. A) The biomass of WT, Δ*cdt-1*, Δ*cdt-2* and Δ*cdt-1*Δ*cdt-2* grown in media with different carbon sources (glucose, cellobiose, xylose, xylodextrin and xylan) at 25°C for 3 days. The mean and standard deviation represent three independent measurements. *P<0.05 (two sided student’s t-test). B) Growth phenotypes of WT, Δ*cdt-1*, Δ*cdt-2* and Δ*cdt-1*Δ*cdt-2* grown in media with different carbon sources (xylan, xylodextrin, Avicel and cellobiose) at 25°C for 3 days. All the media contained Vogel’s salts and 2% various carbon sources. G: glucose, CB: cellobiose, AV: Avicel, X: xylose, XD: xylodextrin, XN: xylan.

Previously, CDT-1 and CDT-2 were identified as cellodextrin transporters [11]. Δ*cdt-2* showed a significant growth defect on cellulose, while Δ*cdt-1* grew similarly to wild-type *N. crassa* [11]. So far, the growth of these *cdt* mutants has only been assessed on media with sucrose and Avicel as carbon sources [11]. To further examine the function of *cdt-1* and *cdt-2* in lignocellulose degradation and utilization, we analyzed the growth of single and double mutants of *cdt-1* and *cdt-2* on different carbon sources (Figure 1). The mutants resulted in no obvious phenotype with glucose or xylose as the carbon source compared with the wild type strain. Under cellobiose growth conditions, the single deletion of *cdt-1* or *cdt-2* has the same phenotype as the wild type strain, but biomass production in the Δ*cdt-1*Δ*cdt-2* strain was only 51% of the wild-type level. No growth of the double knock out strain on Avicel was observed and its conidia did not germinate (data not shown). These results are consistent with previous data that *cdt-1* and *cdt-2* are both cellodextrin transporters, with redundant functions in cellobiose and cellodextrin transport [11]. Surprisingly, the *cdt-2* single deletion strain grew poorly on birchwood xylan, and produced 22% of the biomass of the wild type strain (Figure 1). A growth deficiency was also observed on xylodextrin medium (77% biomass of wild type), but not on xylose (Figure 1). In contrast to the cellulose conditions, the double knock out strain Δ*cdt-1*Δ*cdt-2* showed a similar phenotype as the single deletion strain Δ*cdt-2* under xylan and xylodextrin conditions, particularly for the xylan condition. The observed mutant phenotypes strongly suggest that CDT-2, but not CDT-1, possesses important functions for xylan (hemicellulose) utilization in *N. crassa*. Therefore, we mainly focused on analysis of CDT-2 function during hemicellulose degradation in this study.

### The Efficiency of Xylodextrin Consumption by the Δcdt-2 Mutant is Significantly Lower than WT in *N. crassa*

**Figure 2:**
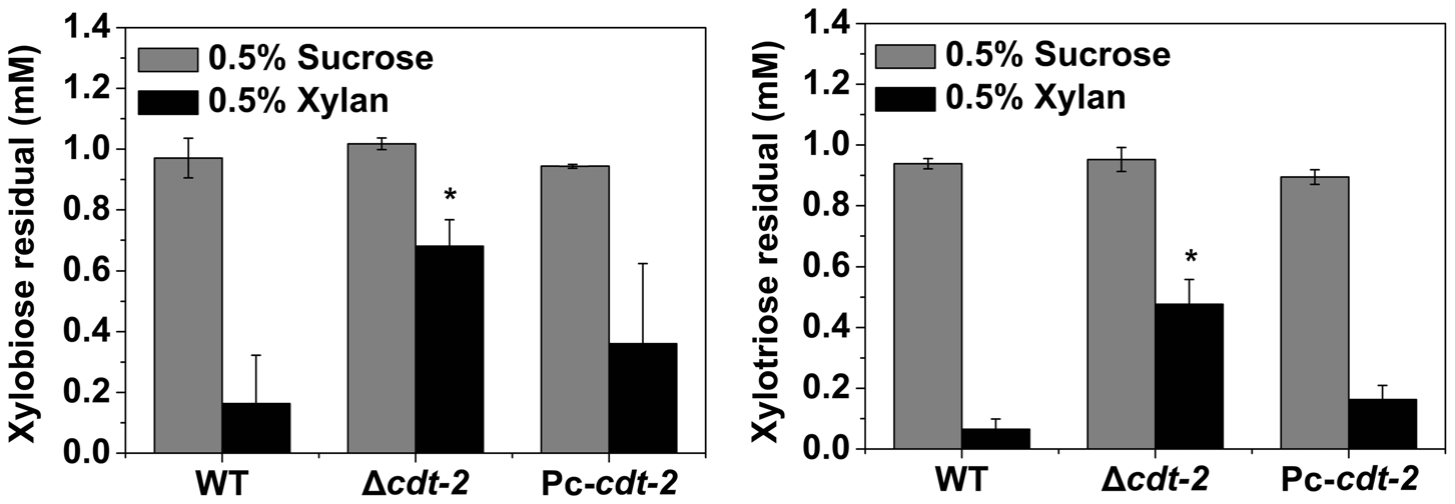
Xylobiose and xylotriose consumption by WT, Δ*cdt-2* and Pc-*cdt-2*. All strains were grown in liquid MM media for 16% xylan or 0.5% sucrose. Ten milliliters of mycelia were collected and incubated with 90 µM of either xylobiose or xylotriose for 15 min. The mean and deviation represent three independent measurements. *P<0.05 (two sided student’s t-test).

The observed growth defect of the *cdt-2* mutant on birchwood xylan suggested that CDT-2 plays a critical role in hemicellulose degradation and utilization. CDT-2 is a cellodextrin transporter, and the cellodextrin consumption capability of Δ*cdt-2* mutants is reduced compared to WT [11]. We hypothesized that the mechanism by which CDT-2 affects cellulose and hemicellulose utilization might be similar. To test this hypothesis, xylobiose and xylotriose consumption assays were performed in Δ*cdt-2* and wild type strains. The consumption assay showed that the xylodextrin consumption of the Δ*cdt-2* strain was significantly reduced compared with wild type (Figure 2). This result suggested that the mode of CDT-2 recognition of hemicellulose and cellulose is similar, and may involve in sensing and/or uptaking of the oligosaccharides cellodextrin or xylodextrin, which are derived from the solid polymers of cellulose and hemicellulose.

### The cdt-1 and cdt-2 Expression Pattern is Different During Growth on a Variety of Carbon Sources

**Figure 3:**
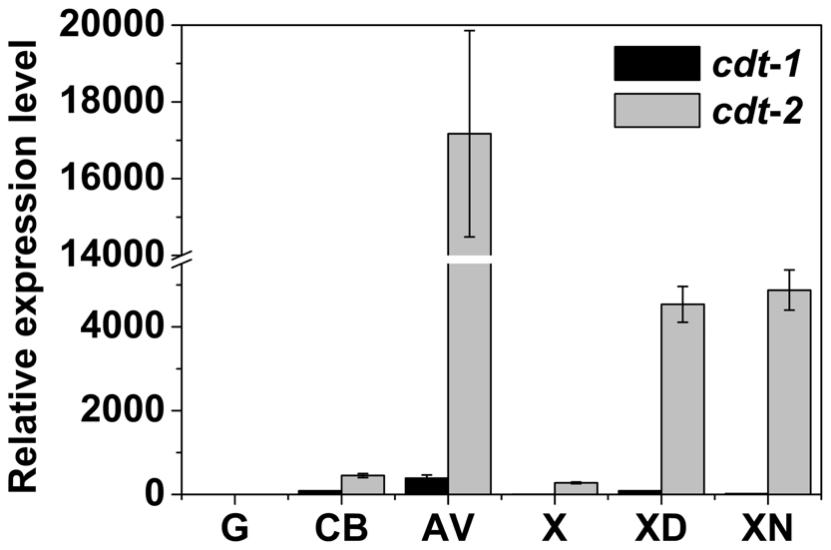
Gene expression level of *cdt-1* and *cdt-2* on different carbon sources. Gene expression levels of *cdt-1* and *cdt-2* on different carbon sources (glucose, cellobiose, Avicel, xylose, xylodextrin and xylan) by qRT-PCR. The wild type strains were grown on different carbon sources at 25°C for 16 h (glucose, cellobiose, xylodextrin and xylan), 22 h (xylose), or 30 h (Avicel). Gene expression levels of actin (NCU04173) were used as an endogenous control in all samples. Each reaction was done in triplicate.

The *cdt-2* mutant had a clear phenotype, but the *cdt-1* mutant was similar to wild type, suggesting differences in the function of these two cellodextrin transporters [11]. In order to further explore the molecular basis of the phenotypes we observed in the two transporter mutants, we investigated the expression pattern of both *cdt-1* and *cdt-2* during growth on several different carbon sources (Figure 3). In general, expression levels of *cdt-2* were higher than *cdt-1*, and *cdt-2* was induced by wider range of substrates (Figure 3, Figure S2 in File S1). Specifically, *cdt-2* was induced to a much higher expression level than *cdt-1* during growth on Avicel, and *cdt-2* expression was induced by xylan and xylodextrin. No significant induction was observed for *cdt-1* under the same conditions. The expression data support the hypothesis that CDT-2 is a critical component in both cellulose and hemicellulose degradation and utilization.

### CDT-2 is Localized to the Cell Membrane during Growth on Both Cellulose and Hemicellulose

**Figure 4:**
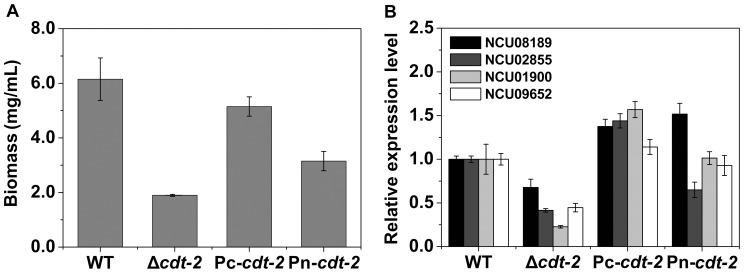
The complementation of *cdt-2* in *N. crassa*. A) The biomass of Pc-*cdt-2* (*his-3*::pMF272-*Pccg-1*-*cdt2*-GFP; Δ*cdt-2*), Pn-*cdt-2* (*his-3*::pMF272-native-*cdt2*-GFP; Δ*cdt-2*), WT and Δ*cdt-2* strains, which were grown on Vogel’s salts medium with 2% xylan as a carbon source at 25°C for 3 days. The mean and standard deviation represent three independent measurements. B) Gene expression levels of hemicellulase genes (NCU08189, NCU02855, NCU01900 and NCU09652) in WT, *cdt-2* mutant, Pc*-cdt-2*, and Pn*-cdt*-2 strains by qRT-PCR. All strains were grown in liquid MM media for 16 h, then transferred into 0.5% xylan for an additional 4 h of cultivation. Gene expression levels of actin (NCU04173) were used as an endogenous control in all samples. Each reaction was done in triplicate.

**Figure 5:**
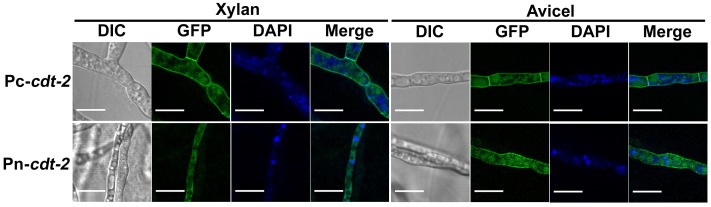
Microscopic observation of CDT-2 subcellular localization in *N. crassa*. Pc-*cdt-2* and Pn-*cdt-2* were grown in liquid MM media for 16 h and transferred into inducing media with Vogel’s salts and either 0.5% xylan or 0.5% Avicel as carbon source at 25°C for an additional 4 h of cultivation. Microscopic observation was performed by a Laser Scanning Confocal Microscope (Leica TCS SP5 II). The nuclei were stained by DAPI. Scale bar = 10 µm.

In order to assess CDT-2 sub-cellular localization in both cellulose and hemicellulose conditions, the *cdt-2* ORF was fused to GFP under the control of either *ccg-1* promoter [31] or its native promoter, and each construct was reintroduced into the *cdt-2* deletion strain. The biomass and xylodextrin consumption defect were partially restored in the resulting strains (Figure 4, Figure 2), and the hemicellulase gene expression phenotype was almost completely restored suggesting a functional GFP-tagged CDT-2 protein. Based on the GFP signal, CDT-2 is primary located in the plasma membrane (Figure 5). Interestingly, we also observed localization of CDT-2 at the septum.

### Transcriptome Analysis of Δcdt-2 on Xylan and Avicel

To further explore the genome-wide effects of CDT-2 during cellulose and hemicellulose degradation and utilization, we performed a transcriptome comparison analysis of Δ*cdt-2* and the wild type strain in response to Avicel and xylan using RNA-seq technology [32], [33] (Table S2 P1).

**Figure 6:**
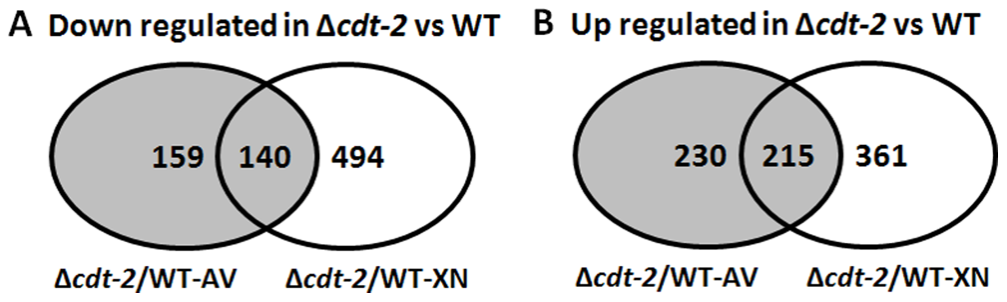
Venn diagram of the transcriptome comparison of WT and Δ*cdt-2* on either Avicel or xylan. A) The genes that show a statistically significant down-regulation in expression level in Δ*cdt-2* compared with WT on xylan or Avicel. Fourteen genes that were down-regulated on both Avicel and xylan in the Δ*cdt-2* strain compared with WT are related to polysaccharide metabolism (Table 2). B) The genes that show a statistically significant up-regulation in expression level in Δ*cdt-2* compared with WT on xylan or Avicel. Funcat analyses of all gene sets are available in the Table S2. The wild type and Δ*cdt-2* strains were grown in liquid MM media for 16 h, then transferred into 0.5% xylan or 0.5% Avicel for an additional 4 h of cultivation.

Consistent with the observed growth defects of the *cdt-2* mutant, 299 and 634 genes were significantly down-regulated in Δ*cdt-2* compared with wild type in response to Avicel and xylan, respectively. A set of 140 genes was down-regulated under both conditions (Figure 6A). In this set of 140 genes, fourteen genes were related to polysaccharide metabolism according to FunCat analysis [34] (Table S2 P2), including seven hemicellulase genes: NCU09923 (β-xylosidase, *gh3-7*), NCU09652 (β-xylosidase, *gh43-5*), NCU08189 (endo-1,4-β-xylanase, *gh10-2*), NCU05159 (acetylxylan esterase), NCU4870 (acetyl xylan esterase), NCU01900 (xylosidase/arabinosidase, *gh43-2*), and NCU00709 (β-xylosidase, *gh3-8*), and other carbohydrate metabolism enzymes (Table 2). Among the down-regulated genes in Δ*cdt-2*, a much larger group of genes (494 genes) were specifically down-regulated on xylan, while 159 were specifically down-regulated on Avicel (Figure 6A). In the group of 494 genes down-regulated on xylan, many genes were not directly involved in xylan degradation, including proteins with binding functions or cofactor requirements (structural or catalytic), metabolism, protein synthesis, and energy (Table S2 P3). This suggests that the *cdt-2* mutation caused a tremendous nutritional challenge for the mutant, leading to the down regulation of many pathways to ensure survival [35]. Thirty one genes in the set of 159 genes down-regulated specifically on Avicel were related to polysaccharide metabolism, containing many cellulase and hemicellulase genes, including several key cellulase genes, such as NCU07340 (cellobiohydrolase-1, *cbh-1*), NCU09680 (cellobiohydrolase-2, *cbh-2*), and NCU01050 (endoglucanase II, *gh61-4*) (Table S2 P4).

**Table 2:** Down-regulated genes related to polysaccharide metabolism in Δ*cdt-2* strain compared with WT on both Avicel and xylan media.

Locus	Gene annotation	RPKM value-AV^b^		Fold change	RPKM value-XN^c^		Fold change
		WT	Δ*cdt-2*		WT	Δ*cdt-2*	
NCU09923	β-xylosidase	34	3	12.1	258	31	8.3
NCU09652	β-xylosidase	694	134	5.2	746	287	2.6
NCU09582	chitin deacetylase	410	3	120.8	44	5	8.4
NCU09175	β-1,3-endoglucanase EglC^a^	1241	657	1.9	780	626	1.2
NCU08755	β-glucosidase 1	1567	404	3.9	170	113	1.5
NCU08384	xylose reductase	2736	280	9.8	8034	1541	5.2
NCU08189	endo-1,4-β-xylanase	8536	860	9.9	4778	2743	1.7
NCU05751	cellulose-binding protein	74	1	67.6	47	14	3.3
NCU05159	acetylxylan esterase	2066	46	45.4	540	108	5.0
NCU04870	acetyl xylan esterase	867	19	46.6	174	32	5.5
NCU01906	aldehyde reductase	136	46	3.0	552	94	5.9
NCU01900	xylosidase/arabinosidase	2023	108	18.8	1689	289	5.9
NCU00709	β-xylosidase	41	6	7.4	229	60	3.8
NCU00130	β-glucosidase	5277	396	13.3	97	52	1.9

a GPI-anchored cell wall β-1,3-endoglucanase EglC.

b Avicel.

c xylan.

In addition, a total of 445 and 576 genes were up-regulated in *cdt-2* deletion strain on Avicel and xylan, respectively, with 215 overlapping genes (Figure 6B). On xylan, 361 genes were specifically up-regulated in the Δ*cdt-2* strain compared with the wild type strain. According to the FunCat analysis, many genes encoding sugar transporters are up-regulated in Δ*cdt-2*, including NCU00821, NCU01132, NCU05853, NCU02188, and NCU04963 (Table S2 P5). These observations suggest that the fungus is responding to the nutrition limitation caused by the *cdt-2* mutation, and this response has some overlap with the response to a no carbon condition [35] (Figure S3 in File S1). The deletion of *cdt-2* also induced a starvation effect under Avicel conditions, but this effect was not as strong as in the xylan condition, suggesting that redundant functions for growth on Avicel exist between CDT-2 and its homolog CDT-1. Interestingly, the ROS (reactive oxygen stress) regulator Nap1 (NCU03905) and one of its target genes, peroxidase (NCU00355) [36], [37], were up-regulated under the Avicel condition, suggesting that ROS stress is generated during cellulose degradation when CDT-2 is deleted (Table S2 P6).

### Overexpression of cdt-2 Increased Cellulase and Hemicellulase Gene Expression in *N. crassa*

**Figure 7:**
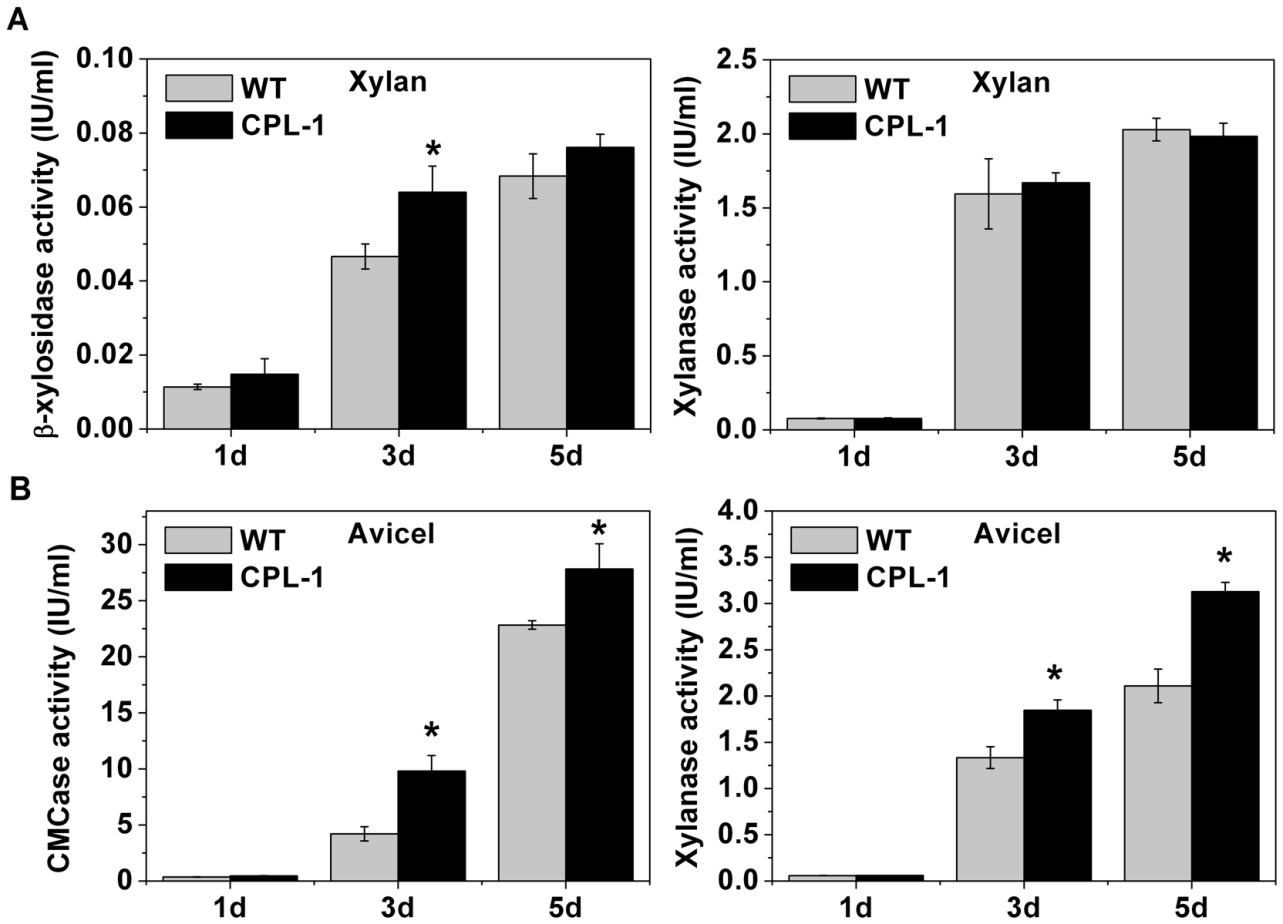
Enzyme activity of culture supernatants of wild type and CPL-1 strains. A) β-xylosidase activity and xylanase activity of wild type strain and *cdt-2* overexpression strain CPL-1 on xylan for 1, 3 and 5 days of cultivation. B) Endoglucanase activity and xylanase activity of WT and CPL-1 on Avicel for 1, 3 and 5 days of cultivation. The mean and deviation showed are three independent measurements. *P<0.05 (two sided student’s t-test).

The deletion of *cdt-2* caused numerous cellulase and hemicellulase genes to be down-regulated, and thus higher cellulase and hemicellulase production may result from *cdt-2* overexpression. We tested this idea by placing *cdt-2* under the *ccg-1* promoter [31], and transformed this construct into the WT background (FGSC 6103). The resulting strain, called CPL-1, contains one extra copy of *cdt-2*. As expected, the major cellulase and hemicellulase genes were up-regulated in the CPL-1 strain under Avicel and xylan conditions, respectively, as determined through qRT-PCR analysis (Figure S4 in File S1). We also measured endoglucanase, xylanase and β-xylosidase activities for 1, 3 and 5 day old CPL-1 cultures. Under Avicel conditions, the activities of all three enzymes in the CPL-1 strain were significantly higher than those in the wild type strain. The β-xylosidase activity was higher in the CPL-1 strain under xylan conditions, and the xylanase activity was not significantly different. As expected, no endoglucanase activity was detected since xylan cannot induce cellulases in *N. crassa* (Figure 7).

### The Regulation of CDT-2 is Different from CDT-1 in *N. crassa*

**Figure 8:**
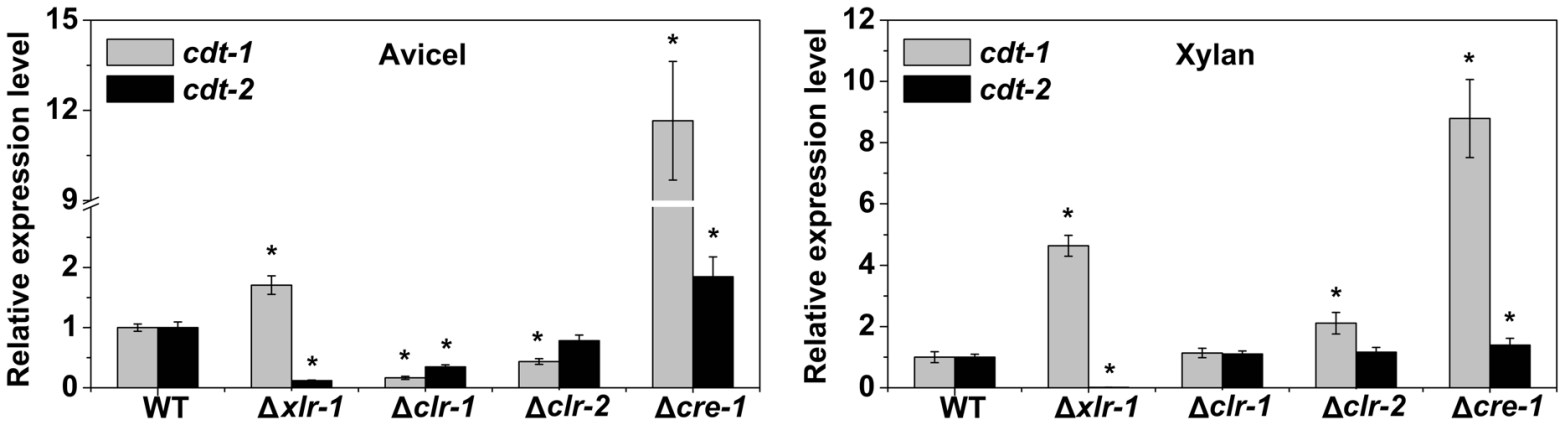
Relative expression levels of *cdt-2* and *cdt-1* in different mutants determined by qRT-PCR. The mutants carrying deletions for different transcription factors (Δ*xlr-1*, Δ*clr-1*, Δ*clr-2* and Δ*cre-1*) were grown in liquid MM media for 16 h, then transferred into 0.5% xylan or 0.5% Avicel for an additional 4 h of cultivation. Gene expression levels of actin (NCU04173) were used as an endogenous control in all samples. Each reaction was done by triplicate. *P<0.05 (two sided student’s t-test).

The transcription factors CLR-1, CLR-2 [16], XLR-1 [19] and CRE-1 [38] are major regulators of cellulose and hemicellulose degradation in *N. crassa*. To determine whether *cdt-1* and *cdt-2* are regulated by these transcription factors during cellulose and hemicellulose degradation, the relative expression level of the two transporter genes was measured by qRT-PCR in deletion mutants of the four transcription factors (Figure 8). The deletion of the cellulose degradation regulator gene *clr-1* significantly affected the expression of both *cdt-1* and *cdt-2* on Avicel. However, *cdt-1* expression was more affected than that of *cdt-2*. Under Avicel conditions, *cdt-1* expression was nearly undetectable without *clr-1* but mildly affected by the deletion of *clr-2*. In the *xlr-1* mutant, *cdt-2* expression was almost completely suppressed under both Avicel and xylan conditions, while *cdt-1* expression was up-regulated under both conditions. These results suggest that XLR-1 is the major regulator for *cdt-2*, whereas *cdt-1* is primarily regulated by CLR-1. Both *cdt-1* and *cdt-2* are up-regulated in a Δ*cre-1* strain on Avicel and xylan, suggesting that CRE-1 does not directly regulate these transporters, which is consistent with the induction of cellulase and hemicellulase through general CCR (carbon catabolite repression) derepression. In summary, the two cellodextrin transporters are regulated differently in *N. crassa* (Figure S5 in File S1).

## Discussion

CDT-1 and CDT-2 are two important cellodextrin transporters in *N. crassa*, and these transporters play critical roles in cellulose degradation [11]. By comprehensive analysis of the two transporters’ gene expression patterns and phenotypes on a variety of carbon sources, including the cellulose and hemicellulose, we found that only CDT-2 is also involved in hemicellulose degradation and utilization. As far as we know, only transporters affecting cellulose (but not hemicellulose) degradation have been reported, including Stp1 and Crt1 in *T. reesei* [12], CdtC and CdtD in *Penicillium oxalicum* [14] and CDT-1 and CDT-2 in *N. crassa* [9], [11]. This is the first time a sugar transporter has been assigned a function in hemicellulose sensing and utilization.

Xylan is a complex and heteropolymeric hemicellulosic polymer, which mainly consists of xylose and arabinose with some glucuronyl, feruloyl, and acetyl groups [39]. Xylan itself is a very powerful inducer for hemicellulase production, and its xylodextrin derivatives can also serve as inducers. Xylobiose or xylooligosacchardie can also induce hemicellulase production, as has been reported in *Aspergillus nidulans* [40], *Thermoascus aurantiacus* [10] and *T. reesei* [41]. The mechanism by which these microbes sense hemicellulose remains unknown.

During the revision of this manuscript, both CDT-1 and CDT-2 were suggested as transceptors for cellodextrin involved in cellulose sensing [42], and here we show evidence of *cdt-2* involvement in hemicellulose sensing and utilization. As a possible transceptor, CDT-2 has a dual function of both transporter and receptor. The mechanisms for sensing hemicellulose might be similar to that of cellulose, through sensing and/or uptaking the putative lignocellulase inducers cellodextrin and xylodextin. Using the yeast system, the preliminary data we had implicated that the CDT-2 might have capability of xylodextrin transport (Figure S6 and Figure S7 in File S1). Currently, how CDT-2 senses cellodextrin, such as structural-based information, is not clear. Similarly, although we know that deletion of *cdt-2* greatly affects hemicellulase synthesis and hemicellulose degradation in *N. crassa*, the detailed mechanism of how the CDT-2 senses xylodextrin requires further experiments.

Although both CDT-1 and CDT-2 were suggested to be cellodextrin transceptors [42], based on our analysis of the differences of CDT-1 and CDT-2, we would suggest that only CDT-2 is a transceptor, and involved not only in cellulose sensing but also in hemicellulose sensing as well. CDT-1 appears to be purely a cellodextrin transporter, based on its expression pattern and lack of growth defects for the *cdt-1* mutant on a variety of tested carbon sources. The cellobiose transport activity of CDT-1 was higher than that of CDT-2, with better maximum velocity (Vmax) in engineered yeast [11]. Considering CDT-2 as a transceptor of cellodextrin and possible xylodextrin, it is a good target protein to start exploring novel components of the lignocellulose sensing pathway.

## Supporting Information

Table S1 Primers used in cloning and quantitative real-time PCR.
(XLSX)

Table S2 Dataset of transcriptome analysis.
(XLSX)

File S1 Includes Figure S1–S7. **Figure S1.** The strain verification of Δ*cdt-1*, Δ*cdt-2*, Δ*cdt-1*Δ*cdt-2* (A) and Pn-*cdt-2*, Pc-*cdt-2*, CPL-1 (B) by PCR. **Figure S2.** The expression kinetics of *cdt-1* and *cdt-2* on Avicel and xylan. Gene expression levels of *cdt-1* and *cdt-2* in WT under different time points. Cultures were inoculated with WT conidia on MM medium for 16 h growth (SU-16 h), on 2% Avicel medium for 30 h growth (AV-30 h), for 2 d growth (AV-2 d) and for 3 d growth (AV-3 d), or on 2% xylan for 1 day (XN-1 d), 2 days (XN-2 d) and 3 days (XN-3 d). **Figure S3.** The transcriptome comparison of Δ*cdt-2* response to Avicel/xylan with WT response to no carbon. A) The genes that showed a statistically differential expression (analyzed by DEGseq, see the detail procedure in method of text) in WT exposed to no carbon compared with Δ*cdt*-2 on xylan, using data of WT on xylan as reference. B) The genes that showed a statistically differential expression in WT exposed to no carbon compared with Δ*cdt*-2 on Avicel, using data of WT on Avicel as reference. The differentially expressed genes and their functions are listed in supplement material (Table S2 P7). **Figure S4.** Relative expression levels of cellulase and hemicellulase genes in wild type and CPL-1 strains A) Relative expression levels of major cellulase genes (NCU07340, NCU09680 and NCU00762) and *cdt-2* (NCU08114) in WT and *cdt-2* overexpression strain CPL-1 on Avicel conditions by qRT-PCR. B) Relative expression levels of major hemicellulase genes (NCU08189, NCU04870 and NCU01900) and *cdt-2* (NCU08114) in WT and CPL-1 strains on xylan conditions by qRT-PCR. All the strains were grown in liquid MM media for 16 h, then transferred into 2% xylan or 2% Avicel for an additional 24 h of cultivation. The actin gene (NCU04173) was used as an endogenous control in all samples. Each reaction was done by triplicate. *P<0.05. **Figure S5.** The regulation of CDT-1 and CDT-2 in *N. crassa*. Both CDT-1 and CDT-2 transport cellobiose and cellodextrin, which degraded from cellulose by cellulase. CDT-2 may transport xylobiose and xylodextrin, which degraded from hemicellulose by hemicellulase. *cdt-2* is primarily regulated by XLR-1. *cdt-1* is mainly regulated by CLR-1 and CLR-2 on Avicel. Besides the primary regulation, there are cross regulation for *cdt-1* and *cdt-2* by CLR-1. **Figure S6.** Hydrolysis of xylodextrin by the intracellular β-xylosidase (GH43-2) and xylodextrin transport by the recombinant *S. cerevisiae* strain. A) SDS-PAGE gel of purified intracellular β-xylosidase (Figure S7). Lane M, protein molecular weight standards (KDa). Lane 1, β-xylosidase after purification over nickel-NTA resin. On the left, the molecular weights (KDa) are shown. B) The hydrolysis activity analysis of purified β-xylosidase^b^ using xylobiose (XB) and xylotriose (XT) as substrates. The data represent the average of three technical replicates with the standard deviation. C) Intracellular D-xylose accumulation of recombinant *S. cerevisiae* strain E(*gh43-2*+*cdt-2*)^a^ containing *cdt-2* and *gh43-2* ^c^. The error bar is the standard deviation of triplicate measurements. ND: not detectable. **Figure S7.** Extracellular and intracellular β-xylosidase activity of the recombinant strain E(*gh43-2*) and the control strain E(423P). No any xylosidase activity was detected in extracellular supernatant of recombinant strain.
(DOCX)
