# Supplementary material for: Evidence of a Critical Role for Cellodextrin Transporte 2 (CDT-2) in Both Cellulose and Hemicellulose Degradation and Utilization in Neurospora crassa
Source: PLoS One. 2014 Feb 20;9(2):e89330. doi: 10.1371/journal.pone.0089330 (PMC3930720; doi:10.1371/journal.pone.0089330)
Supplement: File S1 — Includes Figure S1–S7. Figure S1. The strain verification of Δcdt-1, Δcdt-2, Δcdt-1Δcdt-2 (A) and Pn-cdt-2, Pc-cdt-2, CPL-1 (B) by PCR. Figure S2. The expression kinetics of cdt-1 and cdt-2 on Avicel and xylan. Gene expression levels of cdt-1 and cdt-2 in WT under different time points. Cultures were inoculated with WT conidia on MM medium for 16 h growth (SU-16 h), on 2% Avicel medium for 30 h growth (AV-30 h), for 2 d growth (AV-2 d) and for 3 d growth (AV-3 d), or on 2% xylan for 1 day (XN-1 d), 2 days (XN-2 d) and 3 days (XN-3 d). Figure S3. The transcriptome comparison of Δcdt-2 response to Avicel/xylan with WT response to no carbon. A) The genes that showed a statistically differential expression (analyzed by DEGseq, see the detail procedure in method of text) in WT exposed to no carbon compared with Δcdt-2 on xylan, using data of WT on xylan as reference. B) The genes that showed a statistically differential expression in WT exposed to no carbon compared with Δcdt-2 on Avicel, using data of WT on Avicel as reference. The differentially expressed genes and their functions are listed in supplement material (Table S2 P7). Figure S4. Relative expression levels of cellulase and hemicellulase genes in wild type and CPL-1 strains A) Relative expression levels of major cellulase genes (NCU07340, NCU09680 and NCU00762) and cdt-2 (NCU08114) in WT and cdt-2 overexpression strain CPL-1 on Avicel conditions by qRT-PCR. B) Relative expression levels of major hemicellulase genes (NCU08189, NCU04870 and NCU01900) and cdt-2 (NCU08114) in WT and CPL-1 strains on xylan conditions by qRT-PCR. All the strains were grown in liquid MM media for 16 h, then transferred into 2% xylan or 2% Avicel for an additional 24 h of cultivation. The actin gene (NCU04173) was used as an endogenous control in all samples. Each reaction was done by triplicate. *P<0.05. Figure S5. The regulation of CDT-1 and CDT-2 in N. crassa. Both CDT-1 and CDT-2 transport cellobiose and cellodextrin, which degrad [file pone.0089330.s003.docx]

**Supporting information**

**File S1**

**Figure S1**


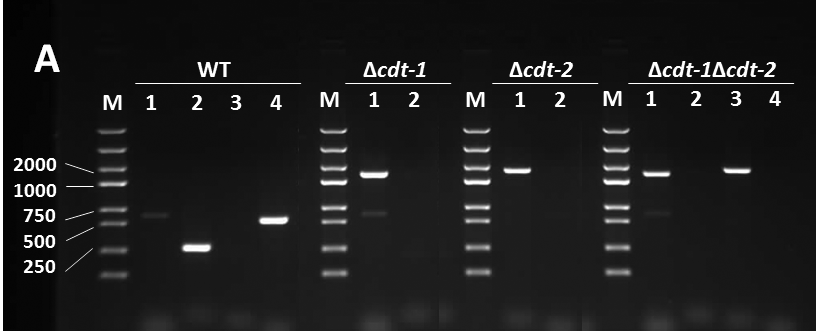


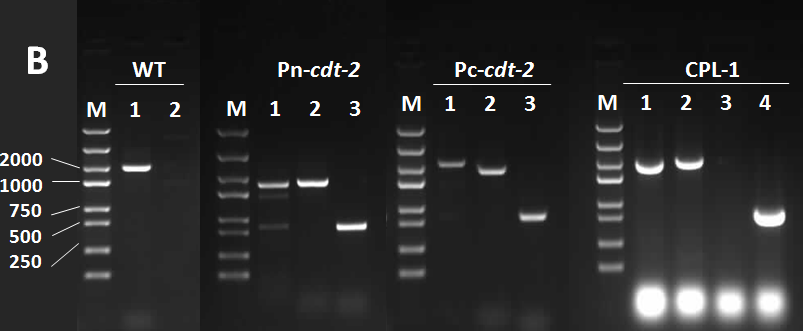


The strain verification of Δ*cdt-1*, Δ*cdt-2*, Δ*cdt-1*Δ*cdt-2* (A) and Pn-*cdt-2*, Pc-*cdt-2*, CPL-1 (B) by PCR.

Figure description:

| Figure A | Location | Primers used | Electrophoretic band size (bp) |
| --- | --- | --- | --- |
| WT | Lane 1 | 801Vr/hph | No band |
|  | Lane 2 | 801VF/801VR | 528 |
|  | Lane 3 | 8114Vr/hph | No band |
|  | Lane 4 | 8114VF/8114VR | 846 |
| Δ*cdt-1* | Lane 1 | 801Vr/hph | 1741 |
|  | Lane 2 | 801VF/801VR | No band |
| Δ*cdt-2* | Lane 1 | 8114Vr/hph | 1857 |
|  | Lane 2 | 8114VF/8114VR | No band |
| Δ*cdt-1*Δ*cdt-2* | Lane 1 | 801Vr/hph | 1741 |
|  | Lane 2 | 801VF/801VR | No band |
|  | Lane 3 | 8114Vr/hph | 1857 |
|  | Lane 4 | 801Vr/hph | No band |
| Figure B | Location | Primers used | Electrophoretic band size |
| WT | Lane 1 | N8114-2-F/N8114-2-R | 1999 |
|  | Lane 2 | C8114G-F/C8114G-R | No band |
| Pn-*cdt-2* | Lane 1 | N8114-F/N8114-R | 1776 |
|  | Lane 2 | 8114Vr/hph | 1857 |
|  | Lane 3 | 8114VF/8114VR | 846 |
| Pc-*cdt-2* | Lane 1 | C8114G-F/C8114G-R | 2081 |
|  | Lane 2 | 8114Vr/hph | 1857 |
|  | Lane 3 | 8114VF/8114VR | 846 |
| CPL-1 | Lane 1 | N8114-F/N8114-R | 1776 |
|  | Lane 2 | C8114G-F/C8114G-R | 2081 |
|  | Lane 3 | 8114Vr/hph | No band |
|  | Lane 4 | 8114VF/8114VR | 846 |

Primers used for strain verification.

| Primer | Sequence |
| --- | --- |
| hph | TGCAATAGGTCAGGCTCT |
| 801Vr | TTAGGGTTGTAGACACCTGC |
| 8114Vr | GACGACCAGAACTAGGTAGG |
| 801VF | CTTATCGTCATGTCCTCCGTCTT |
| 801VR | CGTGCCTCTTTGAGTCTGCTT |
| 8114VF | CGTCTCACCACCATCTACAA |
| 8114VR | CGCAACAGGGTTCGCATA |
| N8114-2-F | CCCAGACAAGACAACATCAT |
| N8114-2-R | GTAGCGTTGGTAAACAGATAGA |
| N8114-F | GACCCAGACAAGACAACATC |
| N8114-R | TCGTGAATCTCCACCTCC |
| C8114G-F | ATCAGCCAACAAAGCAATC |
| C8114G-R | GAAGGTGGTCACGAGGG |

**Figure S2**

**
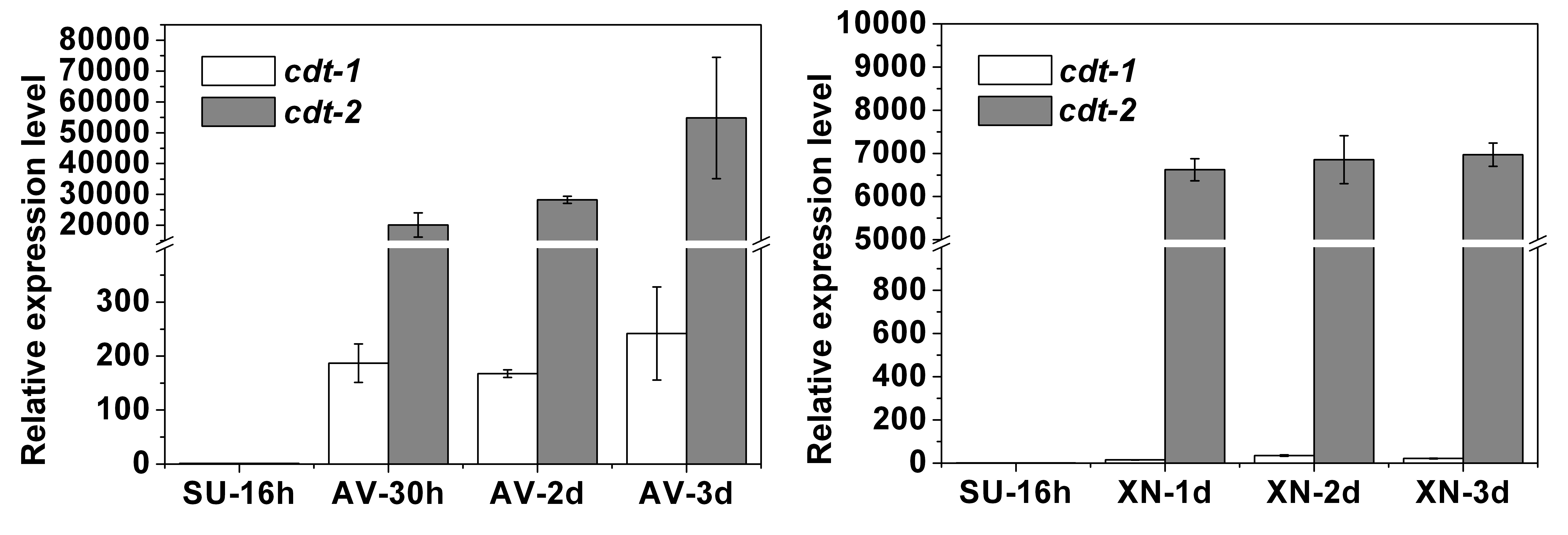
**

**The expression kinetics of CDT-1 and CDT-2 on Avicel and xylan**

Gene expression levels of *cdt-1* and *cdt-2* in WT under different time points. Cultures were inoculated with WT conidia on MM medium for 16 h growth (SU-16h), on 2% Avicel medium for 30 h growth (AV-30h), for 2 d growth (AV-2d) and for 3 d growth (AV-3d), or on 2% xylan for 1 day (XN-1d), 2 days (XN-2d) and 3 days (XN-3d).

**Figure S3**

**
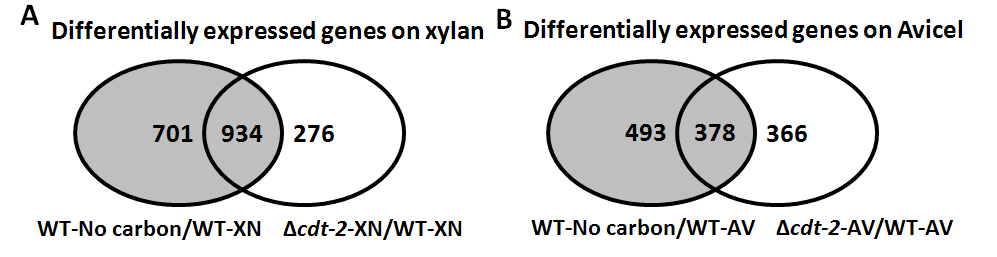
**

**The transcriptome comparison of Δ*cdt-2* response to Avicel/xylan with WT response to no carbon.**

A) The genes that showed a statistically differential expression (analyzed by DEGseq, see the detail procedure in method of text) in WT exposed to no carbon compared with Δ*cdt*-2 on xylan, using data of WT on xylan as reference. B) The genes that showed a statistically differential expression in WT exposed to no carbon compared with Δ*cdt*-2 on Avicel, using data of WT on Avicel as reference. The differentially expressed genes and their functions are listed in supplement material (Table S2 P7).

**Figure S4**


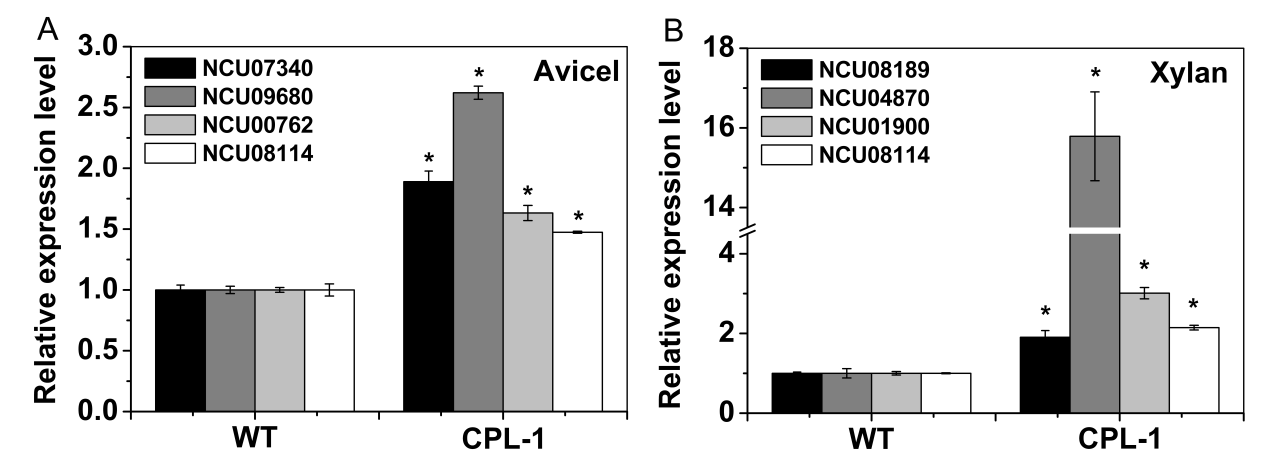


**Relative expression levels of cellulase and hemicellulase genes in wild type and CPL-1 strains** A) Relative expression levels of major cellulase genes (NCU07340, NCU09680 and NCU00762) and *cdt-2* (NCU08114) in WT and *cdt-2* overexpression strain CPL-1 on Avicel conditions by qRT-PCR. B) Relative expression levels of major hemicellulase genes (NCU08189, NCU04870 and NCU01900) and *cdt-2* (NCU08114) in WT and CPL-1 strains on xylan conditions by qRT-PCR. All the strains were grown in liquid MM media for 16 h, then transferred into 2% xylan or 2% Avicel for an additional 24 h of cultivation. The actin gene (NCU04173) was used as an endogenous control in all samples. Each reaction was done by triplicate. *P<0.05.

**Figure S5**

**
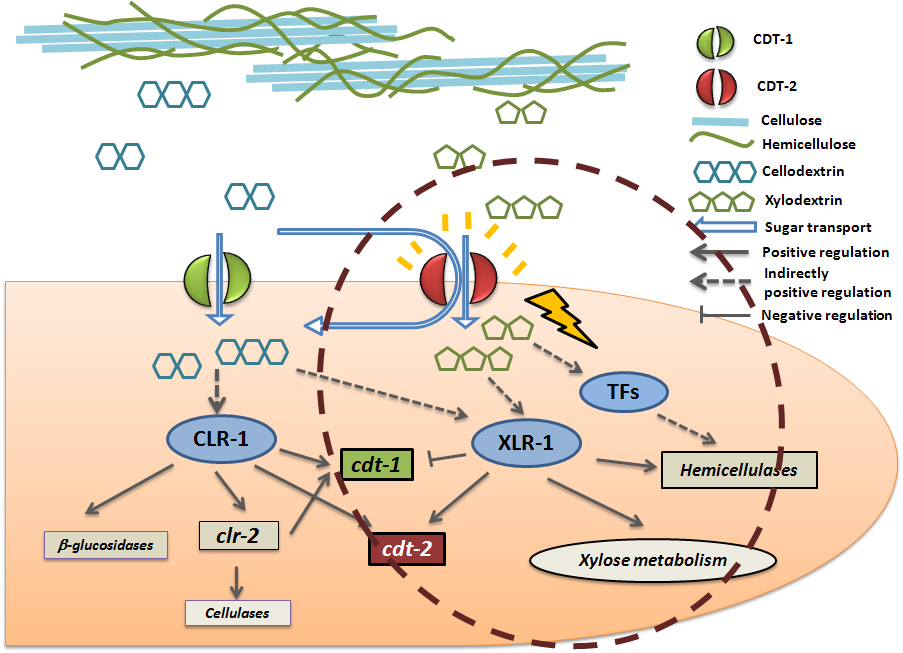
**

**The regulation of CDT-1 and CDT-2 in *N. crassa*.**

Both CDT-1 and CDT-2 transport cellobiose and cellodextrin, which degraded from cellulose by cellulase. CDT-2 may transport xylobiose and xylodextrin, which degraded from hemicellulose by hemicellulase. *cdt-2* is primarily regulated by XLR-1. *cdt-1* is mainly regulated by CLR-1 and CLR-2 on Avicel. Besides the primary regulation, there are cross regulation for *cdt-1* and *cdt-2* by CLR-1.

**Figure S6**





**Hydrolysis of xylodextrin by the intracellular β-xylosidase (GH43-2) and xylodextrin transport by the recombinant *S. cerevisiae* strain.** A) SDS-PAGE gel of purified intracellular β-xylosidase (Figure S7). Lane M, protein molecular weight standards (KDa). Lane 1, β-xylosidase after purification over nickel-NTA resin. On the left, the molecular weights (KDa) are shown. B) The hydrolysis activity analysis of purified β-xylosidase^b^ using xylobiose (XB) and xylotriose (XT) as substrates. The data represent the average of three technical replicates with the standard deviation. C) Intracellular D-xylose accumulation of recombinant *S. cerevisiae* strain E(*gh43-2*+*cdt-2*)^a^ containing *cdt-2* and *gh43-2*^c^. The error bar is the standard deviation of triplicate measurements. ND: not detectable.

**Figure S7**





**Extracellular and intracellular β-xylosidase activity of the recombinant strain E(*gh43-2*) and the control strain E(423P).** No any xylosidase activity was detected in extracellular supernatant of recombinant strain.

**Notes for yeast assay:**

**^a^ Strains and cultures**

The glucose transporter knockout strain *S. cerevisiae* EBY.VW4000 was a kind gift from Prof. Eckhard Boles [[1](#_ENREF_1)]. EBY.VW4000 was grown in YPM media (1% yeast extract and 2% peptone medium containing 2% maltose). *S. cerevisiae* E(*gh43-2*) is a recombinant strain with heterologous expressing the *N. crassa* gene *gh43-2* (NCU01900, β-xylosidase). E(*gh43-2*) was grown in complete minimal dropout media with maltose as the carbon source, supplemented with leucine, uracil and tryptophan. *S. cerevisiae* E(*gh43-2*+*cdt-2*) is a transformed strain expressing *gh43-2* and *cdt-2*. This strain was grown in complete minimal dropout media with maltose as the carbon source, supplemented with leucine and uracil.

Strain and plasmid for xylodextrin hydrolysis and transport experiments in *S. cerevisiae*

| *S. cerevisiae* |  |  |
| --- | --- | --- |
| EBY.VW4000 | CEN.PK2-1C Δ*hxt1-17* Δ*stl1* Δ*agt1* Δ*ydl247w* Δ*yjr160c* Δ*gal2* | [[1](#_ENREF_1)] |
| E(*gh43-2*) | EBY.VW4000, *HIS3*∷pRS423-PGK-*gh43-2* | This study |
| E(*gh43-2*+*cdt-2*) | E(*gh43-2*), *TRP1*∷pRS424-PGK-*cdt2*-GFP | This study |
| E(*gh43-2*+424G) | E(*gh43-2*), *TRP1*∷pRS424-PGK -GFP | This study |
| plasmids |  |  |
| pRS424-PGK-GFP | Yeast multicopy plasmid with PGK promoter and GFP tag | This study |
| pRS423-PGK-*gh43-2* | pRS423-PGK with cDNA of *gh43-2*, *HIS3* | This study |
| pRS424-PGK-*cdt2*-GFP | pRS424-PGK-GFP with cDNA of *cdt-2*, *TRP1* | This study |

**^b^Purification and activity analysis of β-xylosidase (GH43-2)**

The ORF of *gh43-2* (NCU01900) was PCR amplified from *N. crassa* cDNA using primers 1900-F and 1900-R, with 6×His tag added to primer 1900-R. *N. crassa* cDNA was synthesized from mRNA isolated from wild-type *N. crassa* cultured on xylan. Subsequently, *gh43-2* was inserted into plasmid pRS423-PGK using *Spe*I and *Eco*RI restriction sites. The resulting plasmid, pRS423-PGK-*gh43-2*, was transformed into *S. cerevisiae* EBY.VW4000 to construct strain E(*gh43-2*).

One clone of E(*gh43-2*) was inoculated into 50 ml SC-His medium with 2% maltose, and incubated overnight at 30°C. The seed culture was subsequently transferred into one liter of SC-His medium with 2% maltose for 4-6 h of cultivation. The cells were harvested through centrifugation, washed with ice-cold water, and resuspended in PEB buffer: 50 mM Tris-HCl, pH 8.0, 2 mM EDTA and 1×PMSF (added freshly from a 100×stock solution). The cells were lysed using glass beads, and the lysate was cleared through centrifugation at 15000×g for 10 min. The lysate supernatant was bound to Ni-NTA resin using AKTA purifier 10, and washed with wash buffer (buffer A: 0.02 M phosphate buffer, pH 7.8, containing 20 mM imidazole and 0.5 M sodium chloride). The bound β-xylosidase was eluted with elution buffer (Buffer B: 0.02 M phosphate buffer, pH 7.8, containing 300 mM imidazole and 0.5 M sodium chloride). The appropriate fractions were pooled and concentrated using an ultrafiltration membrane with a molecular weight cut-off of 10 KDa (Amicon, Millipore). The protein was frozen in liquid nitrogen, and stored at -80°C. The purity was determined through SDS-PAGE.

The purified β-xylosidase was assayed for hydrolytic activity with xylobiose and xylotriose. The activity was measured after incubating 5 μg of enzyme (1000 ng/μl) with 200 μl of each sugar solution (10 mM). The reactions proceeded for 40 min at 30°C and the hydrolysates were boiled for 10 min to terminate the enzyme action. The protein was removed using an ultrafiltration membrane. The samples were analyzed through HPLC using a HPX-87H column. The peaks were detected with a refractive index detector.

**^c^Xylodextrin transport in recombinant *S. cerevisiae***

The plasmid pRS424-PGK-*cdt2*-GFP was constructed according to previously published methods [[2](#_ENREF_2)] using plasmid pRS424. The plasmids pRS424-PGK-*cdt2*-GFP and pRS423-PGK-*gh43-2* were co-transformed into *S. cerevisiae* EBY.VW4000 using SC-His-Trp plates with 2% maltose as the selection medium. The recombinant strain was designated as E(*gh43-2*+*cdt-2*). The transport of xylobiose or xylotriose was performed according to a previously published method [[3](#_ENREF_3)], with some modifications. The recombinant strain E(*gh43-2*+*cdt-2*) was inoculated into 3 ml SC-His-Trp medium with 2% maltose and incubated overnight. This seed culture was subsequently inoculated into 100 ml medium in a 250-ml shaker flask. After 24 h of growth at 30°C and 250 rpm, the culture was harvested through centrifugation at 4°C with 3500×g and resuspended in SC-His-Trp medium supplemented with 1% xylobiose or 1% xylotriose to a final OD600 of 20. After 4 h of cultivation, one milliliter of the sample was harvested to measure the intracellular sugar concentration. The cells were washed three times with ice-cold water and resuspended in 0.6 ml deionized water. The resulting suspensions were incubated at 37°C with 250 rpm for 2 days to extract the intracellular sugars. After centrifugation and filtering through a 0.22 μm filter, the supernatants were analyzed using HPLC. The xylose and xylitol concentrations were measured by HPLC with a HPX-87H column and refractive index detector according to the manufacturer’s instructions. The sugar uptake was calculated as micromole sugar (xylose and xylitol) extracted per liter of cell culture at OD ~20.

**Supplement reference:**

1. Wieczorke R, Krampe S, Weierstall T, Freidel K, Hollenberg CP, et al. (1999) Concurrent knock-out of at least 20 transporter genes is required to block uptake of hexoses in *Saccharomyces cerevisiae*. FEBS Lett 464: 123-128.

2. Galazka JM, Tian C, Beeson WT, Martinez B, Glass NL, et al. (2010) Cellodextrin transport in yeast for improved biofuel production. Science 330: 84-86.

3. Du J, Li S, Zhao H (2010) Discovery and characterization of novel D-xylose-specific transporters from *Neurospora crassa* and *Pichia stipitis*. Mol BioSyst 6: 2150.
